# Lab-on-a-Chip Magneto-Immunoassays: How to Ensure Contact between Superparamagnetic Beads and the Sensor Surface

**DOI:** 10.3390/bios3030327

**Published:** 2013-09-17

**Authors:** Bernhard Eickenberg, Judith Meyer, Lars Helmich, Daniel Kappe, Alexander Auge, Alexander Weddemann, Frank Wittbracht, Andreas Hütten

**Affiliations:** 1 Department of Physics, Thin Films & Physics of Nanostructures, Bielefeld University, Universitätsstraße 25, 33615 Bielefeld, Germany; E-Mails: jmeyer@physik.uni-bielefeld.de (J.M.); lars@physik.uni-bielefeld.de (L.H.); dkappe@physik.uni-bielefeld.de (D.K.); aauge@physik.uni-bielefeld.de (A.A.); alexander_weddemann@hotmail.com (A.W.); huetten@physik.uni-bielefeld.de (A.H.); 2 Faculty of Arts and Sciences, Chemistry & Chemical Biology, Harvard University, 12 Oxford Street, Cambridge, MA 02138, USA; E-Mail: wittbracht@fas.harvard.edu

**Keywords:** lab-on-a-chip, immuno assay, superparamagnetic beads, granular GMR, microfluidics, biosensors, magnetoresistive sensors, µTAS

## Abstract

Lab-on-a-chip immuno assays utilizing superparamagnetic beads as labels suffer from the fact that the majority of beads pass the sensing area without contacting the sensor surface. Different solutions, employing magnetic forces, ultrasonic standing waves, or hydrodynamic effects have been found over the past decades. The first category uses magnetic forces, created by on-chip conducting lines to attract beads towards the sensor surface. Modifications of the magnetic landscape allow for additional transport and separation of different bead species. The hydrodynamic approach uses changes in the channel geometry to enhance the capture volume. In acoustofluidics, ultrasonic standing waves force µm-sized particles onto a surface through radiation forces. As these approaches have their disadvantages, a new sensor concept that circumvents these problems is suggested. This concept is based on the granular giant magnetoresistance (GMR) effect that can be found in gels containing magnetic nanoparticles. The proposed design could be realized in the shape of paper-based test strips printed with gel-based GMR sensors.

## 1. Introduction

Immuno assays are an established method in medical analysis to determine the concentration of a macromolecular analyte (antigen) in solution [1,2,3]. Recent advances in lab-on-a-chip technologies have made it possible to carry out complete immuno assay procedures on the area of one chip of several cm^2^ [4,5,6]. These, so-called, Micro Total Analysis Systems (µTAS) often employ detectable labels that are connected to an antibody probe. In direct immunoassays, proteins in the sample are immobilized on the chip surface. Labeled antibodies that specifically bind the target antigen are then introduced into the system, binding to the antigens on the surface. In a washing step, unbound labels are removed so that only labels bound to antigen molecules can be measured by a suitable detection scheme. In sandwich immuno assays, only target antigens are immobilized on the surface, which are functionalized with a second antibody. 

Common labels employed in immuno assays are enzymes (ELISA) [7,8,9,10], radioactive isotopes (RIA) [11,12,13], or fluorophores [14,15]. In recent years, however, superparamagnetic beads have attracted interest as alternative labels in immuno assays [16,17]. These beads consist of magnetic nanoparticles that are encapsulated in a polymer shell. When the size of the nanoparticles is below a certain threshold, the superparamagnetic limit, the thermal energy exceeds the magnetic crystalline anisotropy energy of the particles. Typical superparamagnetic limits are on the order of one to several dozen nanometers. Below this limit, the particles exhibit a random, fluctuating magnetization. An ensemble of the particles will therefore not show a net magnetization unless an external magnetic field strong enough to align the magnetic moment vectors is applied [18]. The beads then develop a stray field. Due to this magnetic stray field, the beads can be detected via magneto resistive sensors [19,20,21,22]. These devices offer high-sensitivity detection at low production cost and can even be printed onto standard printing surfaces such as paper using GMR ink [23]. As the output signal is electronic, they are easy to implement into handheld devices. MR sensors currently employed for µTAS detection include spin valves [24,25,26], Hall crosses [27], anisotropic magnetoresistive rings [28], tunnel magnetoresistance (TMR) [22,29], and GMR sensors [30,31,32,33,34,35]. 

Magnetic beads have several advantages compared to other labels. Unlike fluorescent labels, magnetic beads do not bleach, the MR detector can easily be structured onto the chip surface, there is no background signal from the sample, they are safer to handle than radioactive material and the detection is faster than in ELISA. However, the depositing of solved or dispersed particles such as beads onto a surface is usually diffusion limited. Beads, which generally have a low diffusion coefficient due to their comparably large size (primarily, diameters in the range of 100 nm to 10 µm are used for the detection with magneto-resistive sensors), usually settle onto a surface due to gravitational forces only. However, this requires a long time, depending on the size and density of the particles. Polystyrene beads like the Dynabeads M-280 (Invitrogen) with a diameter of 2.8 µm sink at a velocity of 1.7 µm/s when dispersed in water. Thus, sedimentation times in the absence of any flow are on the order of tens of seconds, or even minutes, depending on the system dimensions. Brownian motion, the parabolic flow profile of flows in micro-channels and lift forces [36] exacerbate this problem. As the beads need to contact the sensor surface for binding events between antigens and antibodies to occur, they need to pass the sensor surface at close distance. This issue is aggravated by the fact that microfluidic pumps with a low flow velocity are difficult to design [37,38], making it more difficult for beads to contact the sensor surface before passing the sensing zone.

There exist a few µTAS that work under static conditions, where no microfluidic flow is applied during the time of the binding so that beads can settle on the surface due to gravitational forces. For such devices, repulsive double layer forces may become a problem. Sharp *et al.* [39] demonstrated that a 4.5 µm polystyrene bead might be kept from sedimenting onto an untreated glass surface in 0.5 mM NaCl solution by repulsive forces that act at a distance of 100 nm between bead and surface. Nevertheless, working examples for sedimentation of beads onto a sensor surface have been published. The Naval Research Laboratory in Washington developed a powerful multi-analyte biosensor where beads settle on and bind to functionalized GMR sensors [30,31]. Unbound beads are not removed in a washing step but by a magnetic field gradient. Schotter *et al.* [32] have shown that their GMR sensor offered a sensitivity superior to fluorescence detection at low analyte concentrations. Their experiment, however, required a time step of one hour for the beads to bind to the sensor surface. Koets *et al.* [33] showed that actuation of the bead dispersion during the binding step can decrease the necessary time interval from 30 min to 1.5 min. Thus, an effective method to bring beads into contact with the sensor surface seems necessary to keep assay times down to feasible levels.

Over the decades, different solutions for this problem have been found. These can generally be allocated to one of three categories, one employing magnetic forces, one utilizing hydrodynamic effects and one applying acoustofluidics. In the following paragraphs, these three categories are defined and examples of actual applications are given. However, as these approaches have their disadvantages, a new sensor concept that might solve these problems in the future is presented in the final section.

## 2. Magnetic Approach

**Figure 1:**
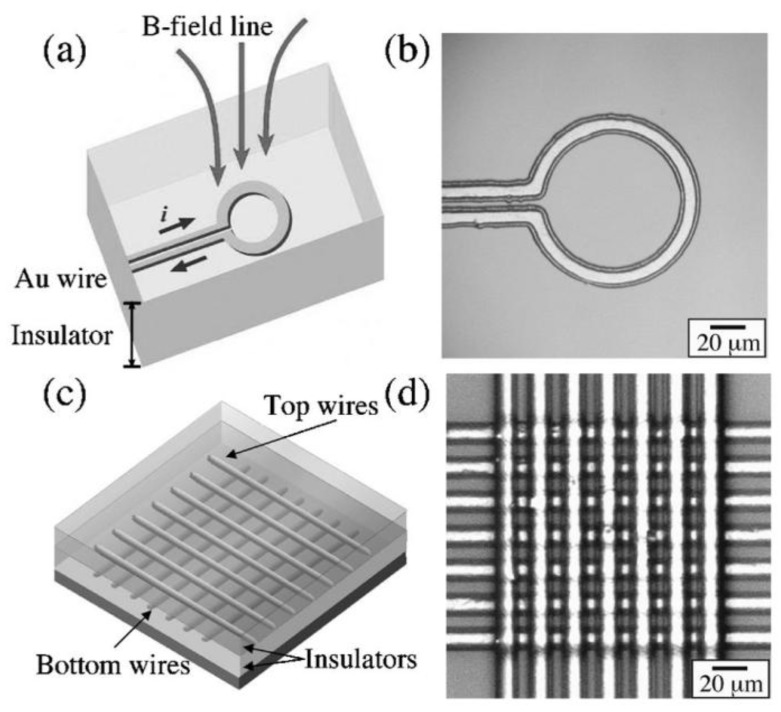
(**a**) Schematic diagram of a microelectromagnet ring trap developed by Lee *et al.* [43] to trap magnetic nanoparticles. (**b**) Micrograph of a fabricated ring trap. (**c**) Schematic diagram of a microelectromagnet matrix which enables the precise movement of clouds of magnetic particles. The matrix consists of two layers of current-carrying conductors with two layers of insulators. (**d**) Micrograph of a fabricated matrix (7 × 7 wires). Reproduced with permission from [43].

The trajectory of superparamagnetic beads flowing in microfluidic systems can be controlled with magnetic fields, e.g., produced by on-chip conducting lines [25,26,29,40,41,42,43]. Thus, one possible approach to ensure contact between the antibody-coated beads and the sensor surface is to employ magnetic field gradients that pull the beads towards the sensor. By adjusting the gradient, the force can be limited to altering the trajectory without fixing unbound beads in place above the sensor. After binding is completed, removal of the magnetic field allows for the detection of the stray fields of the beads on the magneto resistive sensor surface. Such trapping schemes were applied by Graham *et al.* [25] and Lagae *et al.* [26] for spin-valve sensors. Lee *et al.* [43] developed a microelecromagnetic ring trap to capture beads in a small volume with a diameter of 60 µm (see Figure 1(a,b)). Li *et al.* [29] designed a bead concentrator made from current-carrying microstructures that attracts beads and moves them towards a trapping chamber which also serves as the sensing element. This trapping chamber represents a constant volume. When analyte molecules are attached to the beads, their diameter is increased and fewer beads fill the chamber. The underlying TMR sensor then registers the number of beads present in the chamber. This immobilization and detection scheme works best for large biomolecules like DNA. For smaller molecules, additional spacers binding to the analyte are required. 

**Figure 2:**
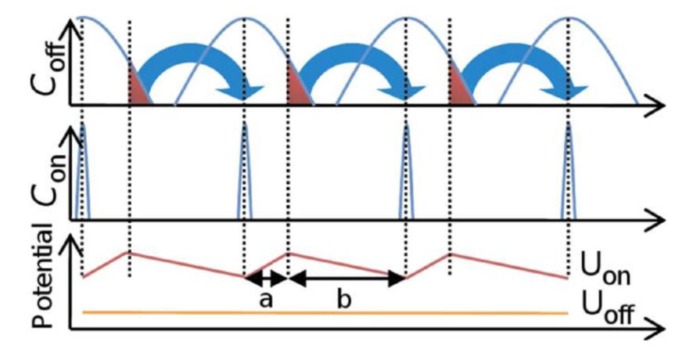
Schematic drawing of the principle of an on-off ratchet built by Auge *et al.* [44]. The concentration distribution C_on_ shows the case that all beads have reached the potential minimum of U_on_. C_off_ shows a concentration distribution after an arbitrary diffusion time in the potential U_off_. The fraction of beads that is successfully transported is marked in red. Reproduced with permission from [44].

However, magnetic fields can be utilized even further. Instead of just assuring contact between bead and sensor surface, they can assist in the transport of beads, rendering microfluidic pumps unnecessary. Lee *et al.* [43] developed a microelectromagnetic matrix made from two layers of current-carrying wires at 90° angle (see Figure 1(c,d)). By changing the magnetic field patterns created by these structures, they were able to control the movement of a particle cloud of 20 µm diameter with high precision. Another method to achieve transport and even separation of different bead species is the construction of a so-called “magnetic on-off ratchet” [24]. In this concept, a magnetic potential asymmetric in time and space combined with non-directional Brownian motion of magnetic beads leads to a net transport of beads in a specified direction (see Figure 2). When the asymmetric field is switched on, beads move to the potential minima until equilibrium between magnetic forces and Brownian motion is reached, resulting in a narrow concentration distribution (C_on_). However, if the fields are switched off, the beads begin to diffuse apart, resulting in a broader concentration distribution (C_off_) after a few seconds of diffusion. When the fields are reactivated, the beads are once again transported to the minima. Due to the asymmetric shape of the potential, a net transport of beads is achieved.

As the diffusion times strongly depend on the hydrodynamic radius of the beads, a separation by size is possible if appropriate time intervals for the switching of the field are chosen. However, the sample size is restricted to objects on the micrometer scale, as magnetic forces are volume dependent and need to overcome the Brownian motion of the particles. On the one hand, objects in the range of a few nanometers show such a strong diffusion that is is not possible to generate strong enough magnetic field gradients within handheld devices. For beads with a diameter above 10 µm, on the other hand, diffusion becomes negligibly small, hindering efficient transport. This problem can be circumvented by modifying the device into a so-called “rocking ratchet” structure [45]. Here, an additional time-dependant force is applied in flow-direction, e.g., in the form of a magnetic field gradient. With an appropriate choice of field strengths and time-dependencies, it is still possible to generate a magnetization dependent net flux of particles without an external bias even at the limit of zero diffusion.

However, utilizing current carrying microstructures to manipulate bead positioning also possesses some disadvantages. First of all, conducting lines have to be structured on-chip and need contacting. Depending on the complexity of the wire structure, this can significantly increase the production costs. To assure that beads contact the sensor surface, conducting lines need to be structured next to the sensors. If they were to be structured right on top of the sensor, an additional layer of insulating material would be required, thus increasing the distance between beads and sensor. This would result in a decreased signal intensity. Thus, the maximum field strength is limited. High field strengths would collect beads right on top of the conducting lines, away from the sensor. Thus, there is a trade-off between field strength (and thus capture range) and resolution. Either the field strength is high and beads in a large liquid volume are attracted but deposit on the conducting lines, or the filtered liquid volume is smaller and the deposition less accurate, leading to more beads being deposited on the actual sensor surface. Additionally, Joule heating caused by the wires might influence sensitive measurements or deteriorate temperature-sensitive analytes.

## 3. Hydrodynamic Approach

**Figure 3:**
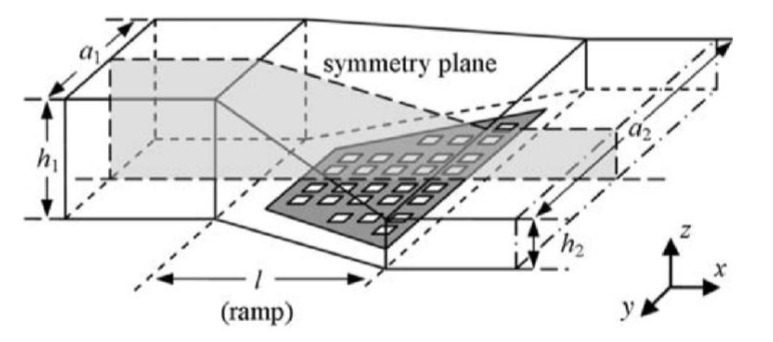
Schematic drawing of the geometry of the ramp structure designed by Weddemann *et al.* [46]. A rectangular microfluidic channel of height *h*_1_ and width *a*_1_ changes over a length *l* into a rectangular channel of height *h*_2_ and width *a*_2_. Particle targets, e.g., a coated sensor array, are placed in the section of decreasing height (ramp). Reproduced with permission from [46].

Instead of employing magnetic fields to draw beads to the sensor surface, hydrodynamic effects caused by variations in the channel geometry can be utilized to support the bead capture process. Weddemann *et al.* [46,47] calculated concentration profiles for bead flows through a rectangular channel, like the one shown in Figure 3. Over length *l* the channel’s height drops from h_1_ to h_2_ and broadens in width from a_1_ to a_2_, forming a ramp-like structure. 

In a straight channel, beads only change their altitude through diffusion and buoyancy. In the ramp structure, the fluid gains an additional motion in the z-direction. The fluid profile drags beads at high altitudes faster towards the sensor surface than beads at low altitudes. This way, Weddemann *et al.* predicted an increase in the capture rate of beads by more than 100%. However, the overall fraction of captured beads still remains small compared to methods that apply the magnetic approach. 

**Figure 4:**
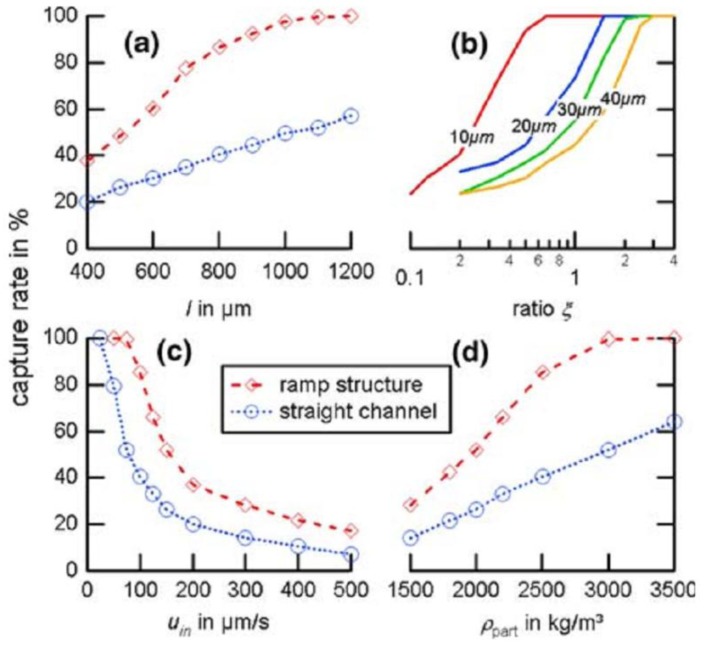
Calculated capture rates of the device presented by Weddemann *et al.* [46] in comparison to a straight channel for different lengths (**a**), cross-section ratios (**b**), inflow velocities (**c**), and particle densities (**d**). If the parameters are not explicitly given, its *l* = 800 µm, *ξ* = 1, *u_in_* = 200 µm/s and *ρ*_part_ = 2,500 kg/m^3^. Reproduced with permission from [46].

Furthermore, while this approach circumvents the problems of producing magnetic gradients by conducting lines, as mentioned above, it increases the complexity of the channel system, as a three-dimensional design is required. Additionally, the whole concept of collecting beads only works for slow velocities up to a few hundreds of µm/s, depending on the remaining parameters (see Figure 4(c)). It is possible to adjust the structure to higher velocities by elongating the sensor surface and by increasing the aspect ratio. However, elongating the ramp to counter higher velocities leads to a dilution of beads captured on the surface as the beads spread over a larger area. This decreases the signal strength and, thus, the sensor’s detection threshold. A higher aspect ratio complicates the manufacturing further, leading to increased production costs.

An alternative approach would be to apply methods of flow focusing utilizing sheath flow. Chiu *et al.* [48] designed a very simple and effective channel system for 3D focusing. However, their design confines the flow to a small area near the center of the channel. For the purpose of bead immobilization on a surface, the focus has to be near the sensing surface. Thus, appropriate modifications of the design would be required, possibly increasing the device complexity.

Although a hydrodynamic approach to the problem seems a reasonable and feasible way to increase binding fractions, few µTAS exist that apply this method. Further research is needed to obtain simple, yet effective structures that have reduced complexity compared to on-chip conducting wires.

## 4. Acoustofluidic Approach

**Figure 5:**
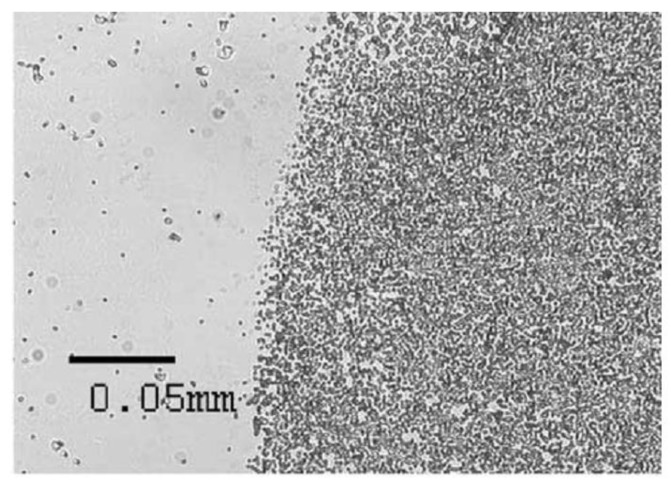
Edge of the ultrasound field that Hawkes *et al.* [51] used to deposit bacteria spores on a functionalized surface. A clear distinction between the deposition area where the field is active (**right**) and the inactive region (**left**) can be seen. Reproduced with permission from [51].

Ultrasonic standing waves constitute another way to move beads onto a sensing surface [49]. An ultrasonic actuator, e.g., a piezo ceramic, can create ultrasonic standing waves inside a microfluidic channel system. Particles inside this standing wave experience radiation forces that depend on the distance between the particle and the nearest pressure node, thus driving them towards these nodes. As the force is proportional to the particle volume, this approach works best for particles in the µm range, e.g., cells or beads. Zourob *et al.* [50] and Hawkes *et al.* [51] used this method to capture *Bacillus subtilis var niger* cells on an activated surface. Hawkes *et al.* reported an efficiency 200 times better than in the absence of the standing wave (see Figure 5). Zourob reported that 96% of the cells were successfully pushed to the surface. Glynne-Jones *et al.* [52] and Oberti *et al.* [53] achieved similar results for beads of 6 µm and 9.6 µm/26 µm diameter, respectively. Using a multi-modal approach which allowed them to switch between an attractive and a repulsive force (facing towards or from the surface), Glynne-Jones *et al.* were even able to remove unfunctionalized beads that were not bound to the surface. Only beads functionalized with streptavidin were left attached to the biotionylated surface. For all of the four systems, operation times were on the order of a few minutes. 

Until recently, acoustofluidic devices were mainly made from metals, silicon, or glass. Gonzalez *et al.* [54] recently presented a chip for the sorting of polystyrene particles of different sizes that was made from SU-8 polymer, while Glynne-Jones *et al.* [55] created a functional chip composed of adhesive transfer tape and cellulose acetate. Thus, a first step towards mass production utilizing polymeric materials has been undertaken. 

The significant advantage of the acoustofluidic approach is fast and efficient collection of particles in the µm range. However, the method is not suitable for smaller particles. Additionally, the systems require miniaturized actuator and reflector layers. To keep production costs down, the actuators have to be built as part of the packaging, and not as part of the chip system. Care has to be taken when combining sensor arrays and ultrasonic standing waves, as the resulting distribution of particles on the surface is often not uniform, but patterned.

## 5. Nanogranular GMR

**Figure 6:**
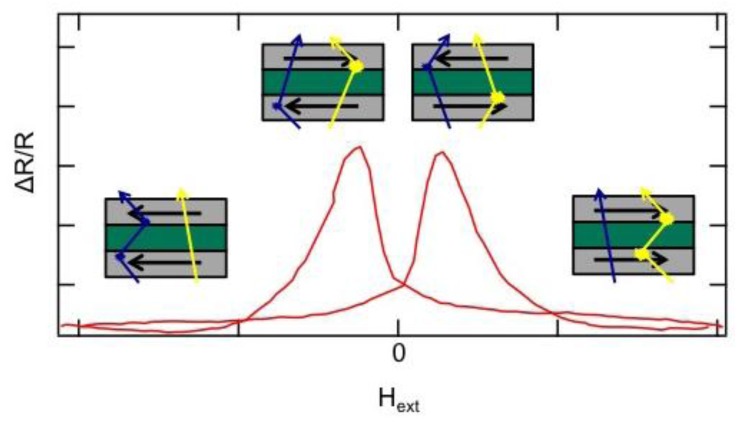
A GMR sensor consists of various layers with a soft fm top electrode which acts as a sensing element and can be manipulated by an external magnetic field. When the magnetization vectors of the layers are parallel, electrons with parallel spin alignment pass through the structure almost without scattering. If the layers are magnetized antiparallel, both electron types are scattered strongly. The resistance of the sensor thus changes if beads, or rather their stray field, influence the magnetization of the upper layer.

As mentioned above, beads can be detected via their stray field by magnetoresistive sensors, e.g., GMR sensors. In these sensors, two ferromagnetic (fm) layers are separated by a third, non-magnetic (nm) layer. The giant magnetoresistance effect (GMR effect) was found and originally studied in magnetic multilayer systems [56,57]. Its physical origin has been explained in terms of spin-dependent scattering of conduction electrons at the interface of the magnetic layers [58]. The scattering probability of electrons passing through the structure strongly depends on the relative orientation of the magnetization of the fm layers (see Figure 6). For a parallel orientation of the magnetization vectors of the fm layers the resistance of the device is low, whereas it is high when the magnetizations are aligned antiparallel. 

**Figure 7:**
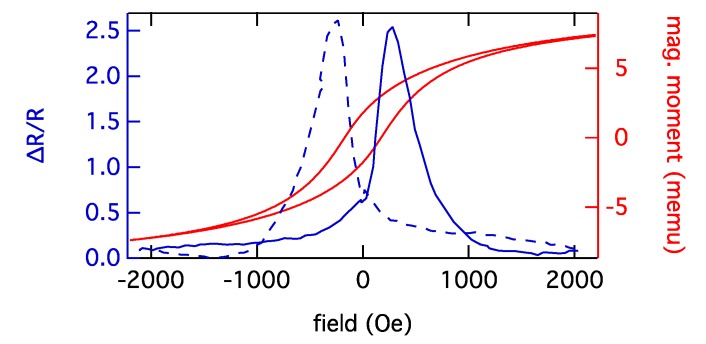
The GMR measurement of a granular system consisting of Co nanoparticles embedded in a gel matrix at room temperature and the corresponding AGM measurement are displayed. The measurement during increasing magnetic field is indicated by the solid- and the measurement during decreasing magnetic field by the dashed line.

However, GMR sensors require well defined surfaces and well controlled lithography. They need to be integrated into a µTAS structure, including microfluidic pumps for low flow velocities. This increases production costs for the chips, often preventing a successful market introduction. However, newly developed printing methods employing GMR ink could change that [23]. As an alternative to GMR ink, granular GMR sensors [59] in the form of GMR gels could be utilized. They are based on the granular GMR effect that was reported in systems consisting of fm granules in metallic matrices [60,61]. Contrary to previous granular systems prepared by sputtering or metallurgical procedures, magnetic nanoparticles can also be integrated into conductive nonmagnetic gel matrices, e.g. salt-containing biogels. For Co nanoparticles, magneto-transport measurements at room temperature revealed GMR effects of more than 200% (see Figure 7), which is far above the values known from common systems [62,63,64]. Regarding technological relevance, this results in enhanced sensor sensitivity.

The possibility of printing gel allows for the realization of granular gel-GMR sensors without employing sputtering or lithography processes. The complexity of the production of such sensors could thus be reduced, leading to a more rapid and less expensive fabrication compared to conventional devices. Besides, biogels such as alginates are abundant in supply and available at low cost. The mechanical flexibility of the matrix might simplify the application in specific geometries or on flexible materials such as paper, thus enabling new lab-on-paper technologies. Finally, the large effect amplitudes will ensure high sensor sensitivity.

**Figure 8:**
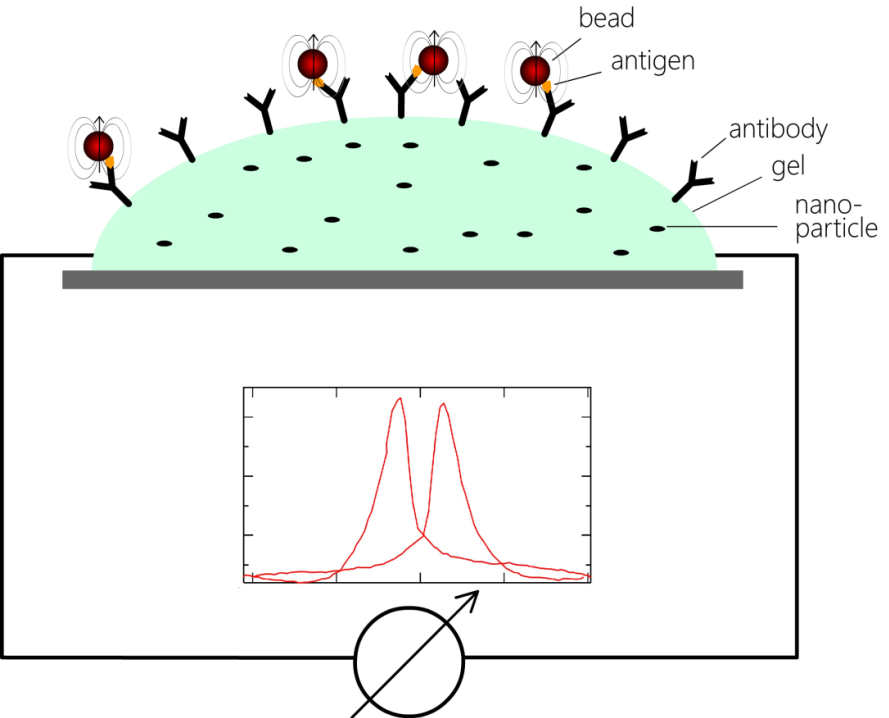
General principle of a gel-based GMR sensor for immuno assays. A droplet of gel containing nanoparticles is deposited on a surface and electrically contacted. Antibodies on the gel surface bind beads that have captured antigens from the solution. The magnetic stray field of the beads influences the resistance of the gel through the granular GMR effect, thus enabling the detection of the concentration of bound beads.

With the help of these gel-based GMR sensors, a new type of paper-based immunoassay µTAS systems could be developed. Instead of structuring sensors on the bottom of a microfluidic channel, spots of GMR gel could be deposited on a small paper strip, similar to a pH test strip. The gel has to contain functional groups that assemble on the surface of the gel and are able to bind antibodies (see Figure 8). Dipping the strip into a specific antibody solution would then activate the strip for a specific sandwich immunoassay. After mixing the test sample (blood, saliva, *etc.*) with a bead solution containing the marker beads and antibodies bound on the bead surface, the activated strip could be dipped into the mixture. Antigens in the solution would link antibodies on the bead surface and antibodies on the gel, thus resulting in beads that are bound to the gel surface. Subsequently to a washing step, the strip would be inserted into a standardized magnetic field. The developing stray field of the beads would alter the resistance of the GMR gel, thus allowing the direct measurement of the bead density on the gel surface. As the mixing takes place under macroscopic flow conditions (no laminar flow), the usual problem of contact between beads and surface could be circumvented. However, repulsive double-layer forces would have to be prevented by careful choice of the surface materials. Due to the simplified fabrication of these strips, the production costs of this µTAS system could possibly be low enough to allow for mass production.
